# Knowledge, Attitudes, and Practices Regarding Typhoid Fever Among University Students in Bangladesh: A Cross‐Sectional Study

**DOI:** 10.1002/hsr2.72774

**Published:** 2026-07-04

**Authors:** Most. Nazma Parvin, Md. Muhtamim Hussain Rifat, Sonia Akther Papia

**Affiliations:** ^1^ Department of Pharmacy Stamford University Bangladesh Dhaka Bangladesh

**Keywords:** Bangladesh, KAP, Typhoid, university students

## Abstract

**Background and Aims:**

Typhoid fever remains a significant public health concern in Bangladesh due to overcrowding, insufficient sanitation, unsafe food and contaminated water exposure, and antimicrobial resistance. The aim of this research is to gather evidence‐based information to guide future public health programs and educational initiatives specifically targeting university students.

**Methods:**

From August 2024 to July 2025, a cross‐sectional survey was conducted among 470 students from three universities in Dhaka city. A structured questionnaire was developed following standard guidelines, and data were collected through convenient sampling. Comparative analyses, Pearson's correlation, multivariable linear regression models, and structural equation modeling (SEM) were used to statistically evaluate the linkage between outcome measures across various sociodemographic and academic variables.

**Results:**

Among 470 valid responses, 127 participants were male (27.02%), and 343 were female (72.98%). Mean ± SD scores were 80.05 ± 16.34 for knowledge, 73.99 ± 24.60 for attitude, and 60.93 ± 16.01 for practice. Knowledge showed weak but significant positive correlations with attitude (*r* = 0.343, 95% CI: 0.261–0.420; *p* < 0.001) and practice (*r* = 0.139, 95% CI: 0.049–0.227; *p* = 0.002), while attitude was positively correlated with practice (*r* = 0.143, 95% CI: 0.053–0.231; *p* = 0.002). In the practice model, knowledge and attitude were not significant predictors after adjustment. Instead, male students demonstrated poorer preventive practices (*β* = −0.448; 95% CI: −0.667 to −0.230; *p* < 0.001), while master's‐level students reported better practices than 1st‐year students (*β* = 0.748; 95% CI: 0.335 to 1.161; *p* < 0.001).

**Conclusion:**

Overall participants in the study had relatively good knowledge and favorable attitudes toward typhoid fever. However, some important gaps remained in preventive practices and vaccination. Behavior‐focused health education, improved access to the typhoid conjugate vaccine, and strengthened campus‐based WASH strategies may help in improving typhoid prevention among university students.

## 1 Introduction

Typhoid fever is a life‐threatening systemic infectious disease caused by the bacterium *Salmonella enterica* serovar Typhi. It is still a major public health concern in low‐ and middle‐income countries where there is insufficient infrastructure for water, sanitation, and hygiene [1]. It affects an estimated 9–12 million people worldwide each year, which causes approximately 128,000 to 161,000 deaths annually [2]. South Asia is still one of the most prevalent areas for typhoid fever [3]. Bangladesh is considered a high‐burden nation due to its dense population, limited access to clean water, and excessive use of antibiotics, which contribute to antimicrobial resistance [4]. Transmission occurs primarily via the fecal‐oral route, often through contaminated water and unsafe street food [5]. Although Bangladeshi households have high access to water, access to safely managed sanitation is still limited, thus increasing susceptibility to infection. Clinical presentation is usually prolonged fever, headache, malaise, and gastrointestinal symptoms, with complications such as intestinal perforation in severe, untreated cases [6, 7]. Accurate diagnosis is still a challenge in resource‐limited settings. Blood culture is the gold standard but requires laboratory capacity, and serological tests such as Widal have poor specificity in endemic regions. The newer rapid diagnostic assays (e.g., Typhidot, Tubex, TPTest) have shown improved performance, especially in children [7, 8].

Antimicrobial resistance further complicates management. Multidrug resistance is decreasing, but fluoroquinolone resistance is widespread, and recent reports of ceftriaxone‐resistant strains highlight an emerging threat [6]. Preventive strategies combining improved water, sanitation and hygiene (WASH) and typhoid conjugate vaccines (TCVs) have demonstrated substantial reductions in disease incidence [9]. With support from Global alliance for vaccines and immunization (Gavi), Bangladesh introduced TCV into its routine immunization program in 2025, alongside enhanced surveillance by national institutions such as the International Centre for Diarrhoeal Disease Research, Bangladesh (icddr,b) and the Institute of Epidemiology, Disease Control and Research (IEDCR) [10, 11].

Despite the high endemicity of typhoid fever in Bangladesh, limited evidence is available on the behavioral determinants of typhoid‐related knowledge, attitudes, and preventive practices among university students. This population is particularly important because students' hygiene, food, water, and healthcare‐seeking behaviors may influence disease transmission on campuses, in households, and in other urban settings. Therefore, this study designed to assess the KAP regarding typhoid fever among university students in Dhaka, Bangladesh and the aim of this research is to gather evidence‐based information to guide future public health programs and educational initiatives specifically targeting university students. The study was guided by a knowledge–attitude–practice (KAP) to behavior perspective, in which knowledge may shape attitudes and, in turn, influence preventive practices. Understanding this pathway is especially relevant in a setting where awareness may not necessarily translate into protective behavior.

## 2 Materials and Methods

### 2.1 Study Design and Participants

From August 2024 to July 2025, a descriptive cross‐sectional study was carried out to investigate the knowledge, attitude, and practices (KAP) regarding typhoid fever among students from three different universities, Stamford University Bangladesh, Jagannath University, and State University of Bangladesh, in Dhaka, Bangladesh. The mentioned inclusion criteria were followed for the design of the study: (1) undergraduate and postgraduate students, (2) participants from multidisciplinary departments, (3) age group ≥18 years, and (4) universities in Dhaka city. Responses with multiple answers for single‐response questions or incomplete responses were excluded from analysis.

### 2.2 Sampling Technique and Sample Size Justification

A convenience sampling technique was used to recruit participants from the selected universities. The sample size was calculated using the single‐population proportion formula for cross‐sectional studies, as described by Lwanga and Lemeshow [12]:

$$n=\frac{Z^{2}p(1-p)}{d^{2}}$$

Where *n* = minimum required sample size, *Z* = standard normal value for a 95% confidence level = 1.96, *p* = expected proportion, and d = margin of error. With a 95% confidence level, 5% margin of error and 50% expected proportion, the minimum required sample size was 385 participants. After adjustment for a possible 10% non‐response or invalid response rate, the required sample size was 422. A total of 470 valid responses were included in the final analysis, which was greater than the minimum required sample size.

### 2.3 Data Collection

Data were collected using two different modes namely Google Forms and one‐to‐one interviews. Google Forms collected data from 194 students, while 276 interviews were conducted by trained data collectors. Participants were informed of the study objectives prior to data collection. Technical terms were explained using standardized explanations when needed to ensure participant understanding. Participation was voluntary, and participants could withdraw at any time without penalty.

### 2.4 Questionnaire Development

The questionnaire was developed based on the World Health Organization (WHO) typhoid fever fact sheet [13], the Centers for Disease Control and Prevention (CDC) resources on typhoid fever and paratyphoid fever [14], and the Bangladesh Ministry of Health and Family Welfare (MOHFW) national guidelines for typhoid management [15].

Before the main data collection, the questionnaire was piloted among 50 university students to test clarity, wording, comprehension, and approximate time needed to complete. Feedback from the pilot test was used to refine the wording of selected items and ensure that technical terms were understandable to participants.

The pilot responses were not included in the final analysis. The final questionnaire included 27 items in 4 groups: sociodemographic information (6 items), knowledge (9 items), attitude‐related items (4 items), and practice‐related items (8 items). For knowledge items, correct responses were scored 1, while incorrect or unclear responses were scored 0. For attitude‐ and practice‐related items, predefined favorable or protective responses were scored 1, while unfavorable, non‐protective, or unclear responses were scored 0. Negatively worded practice items were reverse‐coded so that higher scores consistently indicated better preventive practice.

Domain scores were summed and converted to a 0–100 scale for comparison across KAP domains. Higher scores indicated better knowledge, more favorable attitudes, and better preventive practices. Knowledge scores were classified as poor (< 50) or good (50–100). Attitude scores were categorized as negative (0–40), neutral (41–60), or positive (61–100). Practice scores were categorized as inactive (0–40), moderate (41–60), or proactive (61–100). Internal consistency of the knowledge, attitude‐related, and practice‐related domains was assessed using Cronbach's alpha. Because the items were binary‐coded and covered heterogeneous aspects of typhoid‐related knowledge, attitudes, and practices, the alpha values were interpreted cautiously. Cronbach's alpha values were 0.467 for the knowledge domain, 0.320 for the attitude‐related domain, and 0.337 for the practice‐related domain.

### 2.5 Statistical Analysis

This study is reported according to the Strengthening the Reporting of Observational Studies in Epidemiology (STROBE) statement for observational studies [16]. The statistical analysis was aligned with the Statistical Analysis and Methods in the Published Literature (SAMPL) guidelines [17] and the recommendations for reporting statistics in clinical research described by Assel et al. [18]. The statistical tests used in this study included descriptive statistics, Shapiro–Wilk normality testing, Welch's two‐sample *t*‐test, one‐way ANOVA, Hedges' *g*, eta‐squared, Pearson's correlation, multivariable linear regression, and exploratory SEM. Data management and statistical analysis were performed in R version 4.6.0 using RStudio version 2026.04.0 + 526. The following R‐packages were used as appropriate: stats, readxl, dplyr, broom, ggplot2, psych, effectsize, lavaan, writexl, DiagrammeR, DiagrammeRsvg, and rsvg.

Descriptive statistics were used to describe the characteristics of the participants and KAP scores. Categorical variables were summarized as *n* (%); continuous variables with normal distribution were expressed as mean ± standard deviation (SD). Effect sizes were reported as Hedges' *g* for two‐group comparisons and eta‐squared (*η* ^2^) for comparisons involving more than two groups. Hedges' *g* (approximately 0.2, 0.5, and 0.8) and *η* ^2^ (approximately 0.01, 0.06, and 0.14) values were interpreted as small, medium, and large effects, respectively. Pearson's correlation analysis was used to evaluate the relationship between knowledge, attitude, and practice dimensions, with *r* < 0.4 considered weak, 0.4 ≤ *r* < 0.6 moderate, and *r* ≥ 0.6 strong. Three multivariable linear regression models were fitted separately to identify factors associated with standardized knowledge, attitude, and practice scores. Regression results were reported as standardized regression coefficients (*β*), 95% confidence intervals (CI), and *p*‐values. Exploratory path analysis using structural equation modeling (SEM) was performed with observed composite KAP scores to examine the hypothesized relationships among knowledge, attitude, practice, and participant characteristics. The SEM analysis was considered exploratory because the cross‐sectional design did not allow causal inference. All statistical tests were two‐sided, and a priori significance level of *p* < 0.05 was used.

### 2.6 Ethical Consideration

The study protocol was approved by the Ethical Review Committee of the Faculty of Science, Stamford University Bangladesh, and approval number is SUB/SF/EC‐0701/20. Written informed consent was obtained from all participants prior to data collection, and confidentiality was maintained in accordance with the Declaration of Helsinki [19]. Participants were free to withdraw at any time without consequence.

## 3 Results

Among 470 participants, 127 were male (27.02%), and 343 were female (72.98%). The overall mean ± SD scores were 80.05 ± 16.34 for knowledge, 73.99 ± 24.60 for attitude, and 60.93 ± 16.01 for practice. Majority of our participants (52.55%) belonged to 22–24 years age group, and the dominant number (88.51%) of participants are unmarried. The highest number of participants were 1st year university students (32.13%), and most were living with their parents (59.57%). Nearly two third (64.9%) of the study populations resided urban area. Significant differences in KAP scores were observed across sociodemographic variables. Female participants have higher knowledge scores than male participants (81.8 ± 15.1 vs. 75.4 ± 18.5; *p* < 0.001), with a small‐to‐moderate effect size (Hedges' *g* = −0.393). With a small‐to‐moderate effect size (Hedges' *g* = −0.455), female participants also reported better practice scores than male (62.9 ± 14.7 vs. 55.7 ± 18.2; *p* < 0.001). Education level showed the most consistent differences across KAP domains, with medium effect sizes for knowledge (*p* < 0.001; *η* ^2^ = 0.070), attitude (*p* < 0.001; *η* ^2^ = 0.066), and practice (*p* < 0.001; *η* ^2^ = 0.092). Master's‐level students had the highest knowledge, attitude, and practice scores. Current living arrangement was not significantly associated with knowledge score (*p* = 0.33; *η* ^2^ = 0.007), but it was associated with attitude (*p* = 0.004; *η* ^2^ = 0.028) and practice (*p* = 0.01; *η* ^2^ = 0.024), although both effect sizes were small. Family residence was associated with knowledge (*p* = 0.007; *η* ^2^ = 0.021) and practice (*p* = 0.005; *η* ^2^ = 0.022), again with small effect sizes, while no meaningful difference was observed for attitude (*p* = 0.67; *η* ^2^ = 0.002) (Table 1).

**Table 1:** Comparison of knowledge, attitude, and practice (KAP) scores by participant characteristics among university students in Dhaka, Bangladesh (*N* = 470).

Variables	Parameters	*N* (%)	Knowledge score			Attitude score			Practice score		
			Mean ± SD	*p* value	Effect size	Mean ± SD	*p* value	Effect size	Mean ± SD	*p* value	Effect size
Total	—	470 (100)	80.05 ± 16.34	—	—	73.99 ± 24.60	—	—	60.93 ± 16.01	—	—
Gender	Male	127 (27.02)	75.4 ± 18.5	< 0.001	*g* = −0.393	74.2 ± 26.3	0.91	*g* = 0.012	55.7 ± 18.2	< 0.001	*g* = −0.455
	Female	343 (72.98)	81.8 ± 15.1			73.9 ± 24.0			62.9 ± 14.7		
Age Group	18–21	178 (37.87)	78.5 ± 18.1	0.01	*η* ^2^ = 0.019	66.0 ± 26.9	< 0.001	*η* ^2^ = 0.074	59.8 ± 16.6	< 0.001	*η* ^2^ = 0.038
	22–24	247 (52.55)	79.9 ± 15.5			77.5 ± 22.2			60.0 ± 15.0		
	Above 24	45 (9.58)	86.7 ± 11.0			86.1 ± 17.3			70.5 ± 16.2		
Marital Status	Married	54 (11.49)	85.8 ± 14.5	0.003	*g* = −0.400	79.6 ± 23.8	0.07	*g* = −0.259	61.3 ± 22.7	0.88	*g* = −0.029
	Unmarried	416 (88.51)	79.3 ± 16.4			73.3 ± 24.6			60.9 ± 15.0		
Education Level	1st Year Students	151 (32.13)	80.1 ± 15.8	< 0.001	*η* ^2^ = 0.070	72.0 ± 27.8	< 0.001	*η* ^2^ = 0.066	60.3 ± 15.3	< 0.001	*η* ^2^ = 0.092
	2nd Year Students	118 (25.11)	74.8 ± 18.3			67.4 ± 25.8			57.5 ± 18.8		
	3rd Year Students	77 (16.38)	83.8 ± 13.7			83.1 ± 18.3			61.5 ± 11.1		
	4th Year Students	81 (17.23)	78.7 ± 16.1			72.2 ± 20.5			58.8 ± 13.3		
	Masters	43 (9.15)	89.9 ± 10.8			86.0 ± 16.6			75.6 ± 14.7		
Current Living Places	With Parents	280 (59.57)	81.2 ± 16.7	0.33	*η* ^2^ = 0.007	72.4 ± 25.4	0.004	*η* ^2^ = 0.028	62.0 ± 15.5	0.01	*η* ^2^ = 0.024
	With Relatives	38 (8.09)	77.8 ± 13.9			87.5 ± 25.2			56.9 ± 15.6		
	Hostel/Mess	132 (28.09)	78.5 ± 16.2			72.9 ± 21.6			58.6 ± 16.4		
	Others	20 (4.25)	78.9 ± 15.3			77.5 ± 24.2			68.8 ± 17.4		
Family Residence	Rural	128 (27.23)	76.3 ± 17.3	0.007	*η* ^2^ = 0.021	75.4 ± 26.8	0.67	*η* ^2^ = 0.002	58.6 ± 16.5	0.005	*η* ^2^ = 0.022
	Semi‐Urban	37 (7.87)	79.6 ± 17.9			71.6 ± 27.1			55.4 ± 13.7		
	Urban	305 (64.90)	81.7 ± 15.5			73.7 ± 23.3			62.6 ± 15.8		

*Note:* Hedges' *g* was reported for two‐group comparisons, and eta‐squared was reported for comparisons involving more than two groups. For Hedges' *g*, values of approximately 0.2, 0.5, and 0.8 indicate small, medium, and large effects, respectively. For *η* ^2^, values of approximately 0.01, 0.06, and 0.14 indicate small, medium, and large effects, respectively. *p* < 0.05 was considered statistically significant.

Abbreviations: *g* = Hedges' *g*; *η* ^2^ = eta‐squared; SD = standard deviation.

**Figure 1:**
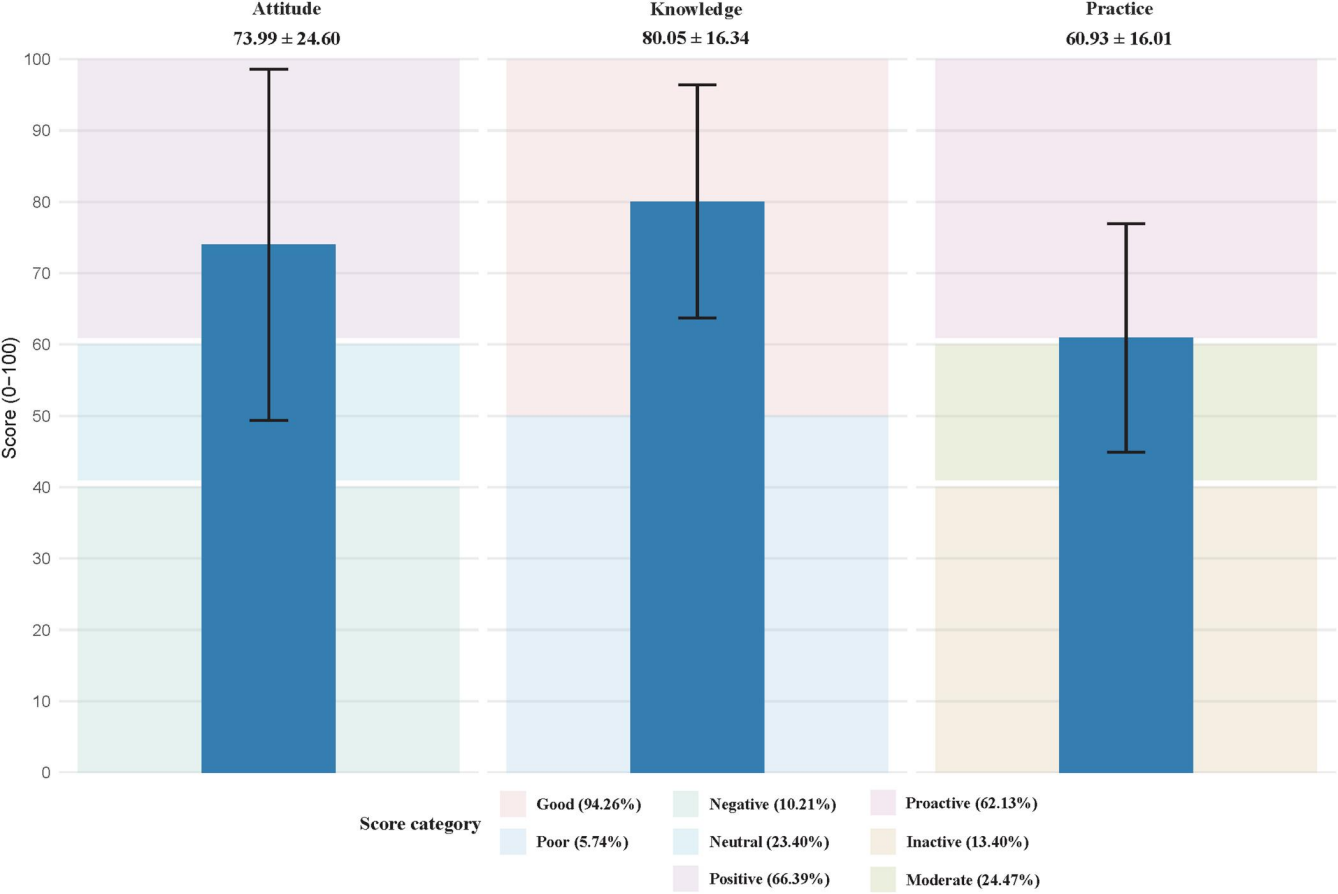
Mean knowledge, attitude, and practice scores with SD error bars and score interpretation bands. Knowledge (9 questions, 0–100 points with <50 as poor level of knowledge and 50–100 as good level of knowledge); Attitude (4 questions with 0–40 as negative, 41–60 as neutral and 61–100 as positive on attitude); and Practice (8 questions with 0–40 as inactive practice, 41–60 as moderate practice, and 61–100 as proactive practice).

The distribution of item‐level responses is presented in Tables 2, 3, 4. Knowledge responses were generally favorable for the food‐ or waterborne nature of typhoid fever (89.15%), bacterial causation (84.68%), contaminated food or water transmission (91.49%), and common symptoms (98.08%). However, comparatively less correct responses were observed for the diagnosis, treatment‐related knowledge, and age‐related response. Attitude‐related responses showed strong awareness of typhoid preventability (89.58%), although responses were less consistent for prevention‐related items. Practice‐related responses showed high reported adherence to hand washing after bathroom use and before meals (98.51%), but lower favorable responses for food‐safety behaviors and typhoid vaccination (25.10%). A high proportion of unclear responses was observed for washing fruits and vegetables before eating, street‐food consumption, and vaccination status. Overall, the study showed that 94.26% of the participants had good knowledge, 66.39% represented positive attitudes, and 62.13% reported proactive preventive practices (Figure 1).

**Table 2:** Distribution of item‐level responses for the knowledge domain, (%).

Qs. no.	Question	Correct	Incorrect	Unclear
K1	Typhoid is a food or water‐borne disease	89.15	3.62	7.23
K2	Typhoid is an illness caused by bacteria due to poor personal hygiene and poor sanitation	93.40	4.26	2.34
K3	We can get typhoid fever by ingesting/eating contaminated food or water	91.49	5.32	3.19
K4	Bacteria is the causative agent of Typhoid fever	84.68	14.47	0.85
K5	Fever, vomiting, diarrhea, & headache are common Signs and symptoms of Typhoid fever	98.08	1.28	0.64
K6	Typhoid can be diagnosed by the Blood Culture Test	71.28	20.21	8.51
K7	Antibiotics are the common treatments for Typhoid fever	60.43	32.77	6.80
K8	Children below 5 are the more prone age group of Typhoid fever	56.80	32.77	10.43
K9	Typhoid fever can cause death if not properly treated	72.77	10.00	17.23

**Table 3:** Distribution of item‐level responses for the attitude‐related domain, (%).

Qs. no.	Question	Favorable	Unfavorable	Unclear
A1	Have you or someone you know ever been diagnosed with typhoid fever?	64.68	21.06	14.26
A2	Do you think typhoid fever is preventable?	89.58	2.55	7.87
A3	Drinking safe water, proper handwashing, & avoiding street food are effective prevention methods.	60.64	26.17	13.19
A4	Have you or someone you know taken antibiotics for typhoid fever?	72.55	6.60	20.85

*Note:* For A1 and A4, responses represent reported experience/history rather than evaluative attitude.

**Table 4:** Distribution of item‐level responses for the practice domain, (%).

Qs. no.	Question	Favorable	Unfavorable	Unclear
P1	Are you concerned about Typhoid fever as a health issue?	73.40	2.56	24.04
P2	Do you drink boiled water?	65.74	17.45	16.81
P3	Do you wash hands with soap and water after using the bathroom?	98.51	0.64	0.85
P4	Do you consume raw milk?	78.08	13.83	8.09
P5	Do you wash your hands before meals?	97.23	0.43	2.34
P6	Do you wash fruits and vegetables before eating?	38.51	0.43	61.06
P7	Do you eat street food?	10.85	36.38	52.77
P8	Are you vaccinated against typhoid fever?	25.10	46.60	28.30

*Note:* For negatively worded practice items, favorable practice indicates the protective response.

Pearson's correlation analysis showed weak but statistically significant positive correlations among KAP domains (Table 5). Knowledge was positively correlated with attitude (*r* = 0.343, 95% CI: 0.261 to 0.420; *p* < 0.001) and practice (*r* = 0.139, 95% CI: 0.049 to 0.227; *p* = 0.002). Attitude was also positively correlated with practice (*r *= 0.143, 95% CI: 0.053 to 0.231; *p* = 0.002).

**Table 5:** Pearson's correlation analysis among knowledge, attitude, and practice scores.

Variable pair	*r*	95% CI	*p* value
Knowledge vs. Attitude	0.343	0.261 to 0.420	< 0.001
Knowledge vs. Practice	0.139	0.049 to 0.227	0.002
Attitude vs. Practice	0.143	0.053 to 0.231	0.002

The results of multivariable linear regression analyses are presented in Table 6. Master's students had higher knowledge scores (*β* = 0.432, 95% CI: 0.012 to 0.852; *p* = 0.04) than 1st‐year students. However, male students were independently associated with lower knowledge scores (*β* = –0.379, 95% CI: –0.599 to –0.159; *p* < 0.001). Similarly, unmarried students (*β* = –0.306, 95% CI: –0.597 to –0.016; *p* = 0.04) and 2nd‐year students (*β* = –0.279, 95% CI: –0.521 to –0.036; *p* = 0.02) had lower knowledge scores. For attitude towards typhoid fever, higher knowledge was a significant positive determinant (*β* = 0.308, 95% CI: 0.222 to 0.394; *p* < 0.001). Older students, both 22–24 years (*β* = 0.443, 95% CI: 0.213 to 0.672; *p* < 0.001) and above 24 years (*β* = 0.742, 95% CI: 0.338 to 1.145; *p* < 0.001) demonstrated more favorable awareness than those aged 18–21 years. In contrast, compared with 1st‐year students, those in 2nd‐year (*β* = –0.273, 95% CI: – 0.501 to – 0.045; *p* = 0.02), and 4th‐year (*β* = –0.361, 95% CI: –0.658 to –0.065; *p* = 0.02) had lower attitude scores. Additionally, living with relatives (*β* = 0.575, 95% CI: 0.243 to 0.907; *p* < 0.001) was positively associated with attitude score, whereas a semi‐urban family residence (*β* = –0.346, 95% CI: –0.682 to –0.010; *p* = 0.04) was negatively associated. In the practice model, knowledge and attitude did not retain statistical significance after adjustment. Instead, male students had lower practice scores (*β* = –0.448, 95% CI: –0.667 to –0.230; *p* < 0.001), while students at the master's level reported better practice than 1st‐year students (*β* = 0.748, 95% CI: 0.335 to 1.161; *p* < 0.001).

**Table 6:** Multivariable linear regression analysis of factors associated with knowledge, attitude, and practice scores.

	*β* (95% CI)	*p* value
Knowledge		
Gender (Male)	−0.379 (−0.599 to −0.159)	< 0.001
Age (22–24)	0.052 (−0.193 to 0.298)	0.68
Age (> 24)	0.285 (−0.146 to 0.716)	0.19
Marital status (unmarried)	−0.306 (−0.597 to −0.016)	0.04
Education level (2nd year students)	−0.279 (−0.521 to −0.036)	0.02
Education level (3rd year students)	0.165 (−0.145 to 0.475)	0.30
Education level (4th year students)	−0.123 (−0.440 to 0.194)	0.45
Education level (masters students)	0.432 (0.012 to 0.852)	0.04
Living place (with parents)	−0.111 (−0.335 to 0.114)	0.33
Living place (with relatives)	−0.091 (−0.446 to 0.264)	0.61
Living place (others)	−0.458 (−0.940 to 0.023)	0.06
Family residence (urban)	0.150 (−0.070 to 0.370)	0.18
Family residence (semi‐urban)	0.095 (−0.265 to 0.454)	0.60
Attitude		
Knowledge	0.308 (0.222 to 0.394)	<0.001
Gender (Male)	0.014 (−0.194 to 0.223)	0.89
Age (22–24)	0.443 (0.213 to 0.672)	<0.001
Age (> 24)	0.742 (0.338 to 1.145)	<0.001
Marital Status (unmarried)	0.075 (−0.197 to 0.348)	0.59
Education level (2nd year students)	−0.273 (−0.501 to −0.045)	0.02
Education level (3rd year students)	0.073 (−0.217 to 0.363)	0.62
Education level (4th year students)	−0.361 (−0.658 to −0.065)	0.02
Education level (masters students)	−0.067 (−0.460 to 0.327)	0.74
Living place (with parents)	−0.082 (−0.292 to 0.128)	0.44
Living place (with relatives)	0.575 (0.243 to 0.907)	<0.001
Living place (others)	0.065 (−0.387 to 0.516)	0.78
Family residence (urban)	−0.083 (−0.289 to 0.123)	0.43
Family residence (semi‐urban)	−0.346 (−0.682 to −0.010)	0.04
Practice		
Knowledge	0.019 (−0.076 to 0.114)	0.70
Attitude	0.084 (−0.012 to 0.181)	0.09
Gender (Male)	−0.448 (−0.667 to −0.230)	<0.001
Age (22–24)	−0.012 (−0.256 to 0.232)	0.92
Age (> 24)	0.388 (−0.041 to 0.816)	0.08
Marital Status (Unmarried)	0.238 (−0.048 to 0.524)	0.10
Education level (2nd year students)	−0.030 (−0.271 to 0.210)	0.80
Education level (3rd year students)	0.111 (−0.193 to 0.415)	0.47
Education level (4th year students)	−0.036 (−0.348 to 0.277)	0.82
Education level (masters students)	0.748 (0.335 to 1.161)	<0.001
Living place (with parents)	−0.084 (−0.304 to 0.136)	0.45
Living place (with relatives)	−0.128 (−0.480 to 0.224)	0.47
Living place (others)	0.179 (−0.294 to 0.652)	0.46
Family residence (urban)	0.079 (−0.137 to 0.295)	0.47
Family residence (semi‐urban)	−0.204 (−0.557 to 0.149)	0.26

*Note:*Reference categories were female sex, age 18–21 years, married marital status, 1st‐year students, hostel/mess living place, and rural family residence. *p* < 0.05 (*), *p* < 0.01 (**), *p* < 0.001 (***).

Abbreviations: β = standardized regression coefficient; CI = confidence interval.

**Figure 2:**
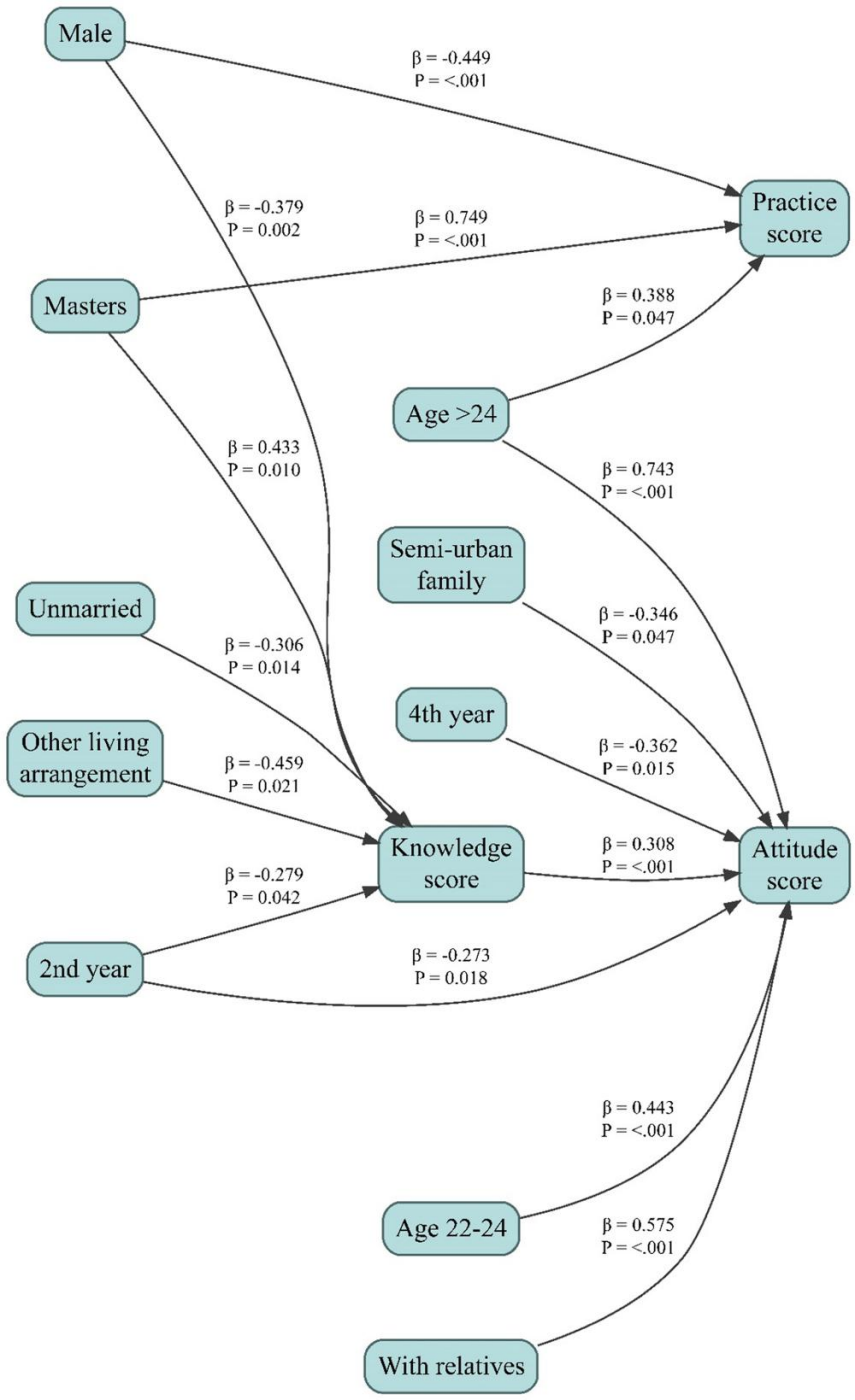
Exploratory structural equation model showing standardized path coefficients and *p*‐values for factors associated with knowledge, attitude, and practice scores.

The exploratory structural equation model (SEM) findings were broadly consistent with the multivariable regression results (Figure 2). Male students were negatively associated with both knowledge (*β* = –0.379, *p* = 0.002) and practice (*β* = –0.449, *p* < 0.001) scores. Knowledge was positively associated with attitude (*β* = 0.308, *p* < 0.001) but neither knowledge nor attitude had a significant direct effect on practice. Students aged above 24 years were positively associated with attitude (*β* = 0.743, *p* < 0.001) and practice (*β* = 0.388, *p* = 0.047). Master's students had higher knowledge (*β* = 0.433, *p* = 0.010) and practice scores (*β* = 0.749, *p* < 0.001), whereas 2nd‐year students had lower knowledge (*β* = –0.279, *p* = 0.042) and attitude scores (*β* = –0.273, *p* = 0.018).

## 4 Discussion

This study showed that students from selected universities had relatively good knowledge and generally positive attitudes toward typhoid fever, but important gaps remained in preventive practices. Knowledge score demonstrated that students are aware of the causes and symptoms of the typhoid fever. However, the awareness of typhoid fever is not fully translated into its treatment, risk groups, and therefore, prevention remained less consistent.

The overall pattern of knowledge and attitude observed in this study is broadly comparable with, and in some areas more favorable than that reported in several community‐based studies. A survey in Pakistan reported that 93.3% of participants were familiar with typhoid fever [20], while a study among food handlers in Lagos, Nigeria, reported approximately 90% favorable attitudes toward typhoid fever [21]. However, research in highly resource‐limited settings such as refugee population in Burundian has demonstrated substantially lower levels of knowledge regarding transmission and prevention of typhoid [22].

The comparatively favorable findings in the present study may reflect the university student's greater educational exposure, and better access to health information. Most participants had good knowledge and positive attitudes toward typhoid prevention. But the lower practice score demonstrated that these findings are not interpreted in preventive measures accordingly. Therefore, the study emphasizes an important gap between knowledge and practice, particularly in treatment, vaccination uptake, and food‐safety practices.

This study suggests that knowledge alone may not be sufficient to motivate protective behaviors. University students may consume street foods or shared food sources because those are inexpensive, convenient, readily available, and socially acceptable. Eating behaviors may also be influenced by social and cultural behaviors such as group eating and patronage of local food vendors. Participants may be aware of the importance of safe food and water but unable to act on their knowledge due to cost and time, campus food environment, perceived convenience, and limited control over food preparation. Some of the responses to the practice items were somewhat ambiguous, suggesting some uncertainty or inconsistency in behaviors, especially with respect to washing fruits and vegetables, street food, and vaccination status. Having good knowledge and fair attitudes, but vaccine uptake was low. Such factors may relate to a perception of low personal risk, limited awareness of vaccine availability, barriers to cost or access, or lack of specific vaccination opportunities for older adolescents and young adults in university settings. Because typhoid fever is common in endemic settings, some participants may underestimate their personal risk or view the disease as treatable rather than preventable. Bangladesh's recent introduction of a typhoid conjugate vaccine into routine immunization may not have reached university‐aged students through childhood vaccination programs. Therefore, university‐based vaccination awareness, counseling, and catch‐up strategies may be useful for improving protection in this population.

The correlation and exploratory SEM findings further support the presence of a knowledge‐practice gap. Knowledge was positively associated with attitude, but neither knowledge nor attitude showed a statistically significant direct association with practice after adjustment. This suggests that increased knowledge may influence attitudes. However, additional intervention components may be necessary to change behavior to address practical barriers, perception of risk, vaccine availability, cultural food practices, and food and water safety at the campus level. Therefore, typhoid prevention strategies should not only disseminate information but should also include behavior‐ and environment‐centered interventions.

Furthermore, the male participants scored significantly poorer in knowledge and practice, suggesting the need for gender‐sensitive health promotion strategies. The observed sex difference may be attributed to differential exposure to health information, perceived susceptibility, hygiene practices or dietary preferences. Participants with a master's level of education had higher scores on knowledge and practice scores, possibly due to greater academic maturity, exposure to health‐related information, and better health‐related decision‐making capacity. These findings have several public health implications. Interventions should focus on food safety education, food vendor surveillance on campus, promotion of safe drinking water, and practical reminders for improving hand hygiene and food safety practices. Targeted interventions for male students, younger students, and students from a semi‐urban or rural background may help to bridge the gaps in KAP outcomes and promote the use of preventive strategies.

Overall, this study provides evidence on typhoid‐related KAP among university students in Bangladesh, and it shows that adequate knowledge does not always translate into effective practice for typhoid prevention. Bridging this gap will require a combination of educational, behavioral, environmental, and vaccination approaches, particularly in university settings where food, water, hygiene, and healthcare‐seeking practices are key determinants of typhoid prevention.

## 5 Conclusion

This study showed that university students in Dhaka, Bangladesh, had relatively overall good knowledge and positive attitudes toward typhoid fever; however, essential preventive practices, particularly vaccination uptake and food‐safety behaviors, remained suboptimal. KAP scores varied according to gender, age, education level, and living conditions. These findings support the need for targeted, behavior‐focused health education, improved access to typhoid conjugate vaccine (TCV), and strengthened campus‐based preventive strategies to reduce typhoid risk and support antimicrobial resistance control.

## 6 Limitations

This study has several limitations. First, the participants were drawn from only three universities in Dhaka, Bangladesh, and excluding students from other universities, other cities, and young adults not attending university, which limit generalizability to the broader population. Second, the cross‐sectional design of this study precluded causal inference among knowledge, attitudes, and practices. Third, the use of self‐reported data, collected through Google Forms and in‐person interviews, might have introduced both recall bias and social desirability bias. Fourth, the internal consistency of the KAP domains was low, possibly due to the heterogeneity of the items, dichotomous scoring, and the inclusion of different dimensions of typhoid‐related knowledge, attitudes, and practices. Therefore, the domain scores should be interpreted cautiously. Finally, using both online and interviewer‐led methods to collect data might have affected how participants answered the questions.

## 7 Future Recommendations

Future research should use multicenter, larger samples sizes from various universities and geographical regions to improve generalizability. Longitudinal and mixed‐methods designs may help clarify causal pathways and explore the knowledge–practice gap in greater depth. Future studies should incorporate objective measures such as validated vaccination records and explore barriers related to access and affordability to typhoid prevention. Subgroup analyses by gender, academic department, university, and residence may help to identify differential KAP patterns. In addition, future interventions should combine behavior‐centered health education with improved access to typhoid vaccination and enhanced campus WASH policies. It is also suggested that KAP survey tools be improved and psychometrically validated to reduce measurement bias.

## Author Contributions

Most. Nazma Parvin conceived, supervised, and contributed to the data collection of the study. Md. Muhtamim Hussain Rifat contributed to data collection, data curation, and drafting of the manuscript, and Sonia Akther Papia contributed to drafting of the manuscript. All authors contributed to study design, interpretation of the findings, critical revision of the manuscript, and approval of the final version.

## Funding

This research received no specific grant from any funding agency in the public, commercial, or non‐for‐profit sectors. No external funder had any role in study design; data collection, analysis, interpretation of the data; writing of the report; or the decision to submit the manuscript for publication.

## Ethics Statement

The Ethical Review Committee of the Faculty of Science, Stamford University Bangladesh approved the study protocol (SUB/SF/EC‐0701/20).

## Author Declaration

All authors have read and approved the final version of the manuscript. Most. Nazma Parvin, the corresponding author, had full access to all of the data in this study and takes complete responsibility for the integrity of the data and the accuracy of the data analysis.

## Transparency Statement

Most. Nazma Parvin affirms that this manuscript is an honest, accurate, and transparent account of the study being reported; that no important aspects of the study have been omitted; and that any discrepancies from the study as originally planned have been explained.

## Data Availability

The data that support the findings of this study are not publicly available because they contain information that could compromise the privacy of research participants. De‐identified data may be made available from the corresponding author upon reasonable request, subject to approval by the relevant ethics committee.
